# Functional and structural characteristics in patients with diabetic macular oedema after switching from ranibizumab to aflibercept treatment. Three year results in real world settings

**DOI:** 10.1186/s40942-022-00373-5

**Published:** 2022-04-01

**Authors:** Anastasios E. Sepetis, Holly Clarke, Bhaskar Gupta

**Affiliations:** Department of Ophthalmology, University Hospital Southampton NHS Foundation Trust, Tremona Road, Southampton, SO16 6YD Hampshire UK

**Keywords:** Aflibercept, Anti-VEGF, Diabetic macular oedema, Ranibizumab, Treatment switch

## Abstract

**Background:**

Our aim was to examine the long term anatomical and functional outcomes in patients with refractory diabetic macular oedema (DMO) undergoing treatment switch from ranibizumab to aflibercept.

**Methods:**

Retrospective review of patients with DMO undergoing treatment switch from ranibizumab to aflibercept at a single centre between 2015 and 2017. Primary outcomes were best corrected visual acuity (BCVA) and central macular thickness (CMT).

**Results:**

57 eyes from 44 patients were included. Following switch to aflibercept, median (IQR) BCVA improved to 73 (64–77) letters at 3 months (*p* = 0.0006), to 73 (61–78) letters at 6 months (p = 0.0042), to 73 (65–77) at 9 months (*p* = 0.0006), and to 73 (63–75) letters at 18 months (p = 0.0444). At 36 months following switch, 12 eyes had gained > 10 letters, 5 eyes had gained 5–9 letters, 25 remained stable (± 5 letters), 7 eyes lost 5–9 letters and 8 eyes lost > 10 letters. A significant reduction in CMT at all trimesters following treatment switch was found except at month 24.

**Conclusions:**

We provide real world data suggesting a sustained anatomical and functional benefit of switching from ranibizumab to aflibercept in the treatment of refractory DMO.

**Supplementary Information:**

The online version contains supplementary material available at 10.1186/s40942-022-00373-5.

## Introduction

Diabetic Macular Oedema (DMO) is the leading cause of visual loss in diabetic patients, creating a significant burden for healthcare services [1]. The National Institute for Clinical Excellence (NICE) currently recommends anti-VEGF injections (ranibizumab or aflibercept) as first line treatment in patients with DMO and increased central retinal thickness (CRT) > 400 µm at the start of treatment, with associated visual impairment [2, 3].

Although the efficacy of ranibizumab in treating DMO has been widely validated, there appears to be a subset of patients with a suboptimal response [4–7]. Common clinical practice in this situation is to switch treatment from ranibizumab to aflibercept. There is only a limited evidence base to support this practice. Several small studies have demonstrated anatomical and functional improvement following treatment switch in DMO patients from ranibizumab or bevacizumab to aflibercept [8–11], with a limited follow-up duration ranging from 1 to 6 months. In contrast, other small studies suggest anatomical improvement only, with no significant change in visual function following treatment switch to aflibercept [12–15].

A sustained benefit was suggested by McCloskey et al. with both anatomical and visual gains reported up to 24 months following treatment switch from ranibizumab to aflibercept in a small retrospective case series of 18 eyes with DMO [16].

Our aim was to examine the long term anatomical and functional outcomes in patients with DMO at 3 years following treatment switch from ranibizumab to aflibercept in a larger cohort of patients.

## Methods

A retrospective review of electronic patient records was carried out at a single tertiary centre at Southampton General Hospital. Patients included in the study had been diagnosed with clinically significant macular oedema secondary to either Type 1 or Type 2 Diabetes Mellitus and had switched from ranibizumab (0.5 mg/0.05 ml) to aflibercept (2 mg/0.05 ml) between 2015 and 2017. All eligible eyes which are treatment naïve to anti-VEGF injections receive fixed 5 monthly injection (ranibizumab/aflibercept) as a loading dose and then treatment and extend (TAE). When patients are switched to alternate therapies its treat and extend with no defined loading dose, it is at discretion of treating physician. However, as these are real world data there are occasions of deviation from the standard protocol either due to patient’s non-attendance or capacity issues.

Primary outcomes were best corrected visual acuity (BCVA) at each visit and maximum central macular thickness (CMT) within 3 mm from the fovea, as recorded on spectral domain optical coherence tomography (OCT). Secondary outcomes included the total number of injections, the number of aflibercept injections following treatment switch, and treatment related adverse events. Data was recorded at 3 month intervals starting from 6 months prior to treatment switch, up to 36 months following the switch.

### Exclusion criteria

Patients treated with less than 3 injections of ranibizumab prior to switching to aflibercept were excluded, as well as those receiving their last injection of ranibizumab more than 120 days before commencing aflibercept. Any patients treated with intravitreal steroid therapy during the study period, and those with multi-factorial macular oedema (e.g. concurrent retinal vein occlusion) were also excluded.

### Statistical analysis

All statistical tests were performed using GraphPad Prism (Version 8.4.1, GraphPad Software, LLC). Gaussian distribution was tested for all values using the D’ Agostino and Pearson test. Where the normality test was not passed, median values and interquartile ranges (IQR) are reported, otherwise mean and range (minimum–maximum) were used. Two-tailed Wilcoxon matched-pairs signed rank test was used for comparison between groups if the normality test was not passed and paired t-test was used for values with Gaussian distribution. For all analyses, a p-value of < 0.05 was considered to be statistically significant. Missing values were not included.

## Results

### Baseline characteristics

Baseline characteristics of the patients are summarised in Table 1. We identified 57 eyes from 44 patients (30 were males) that were eligible. The mean age of patients was 64.4 (41–79) years. The majority of patients had Type II Diabetes mellitus (39 out of the 44) and the remaining 5 had Type I Diabetes mellitus. 45 eyes were phakic at the date of the treatment switch. At the point of switching from ranibizumab to aflibercept, the median BCVA (IQR) was 69 (62–74) ETDRS letters (LogMAR 0.32) and median CMT (IQR) was 400 (358–451) μm. They had received a mean number of 12 (3–21) injections of ranibizumab over a mean period of 17.9 (2.7–42.3) months. The mean period between the last injection of ranibizumab and the first injection of aflibercept was 62.9 (28–119) days. Prior to treatment with ranibizumab, 28 eyes had received focal/grid laser photocoagulation, 17 eyes had received panretinal photocoagulation (PRP) and 5 eyes had received intravitreal triamcinolone. During treatment with ranibizumab, 6 eyes received focal/grid laser, 9 eyes PRP, and 1 eye underwent pars plana vitrectomy (PPV).

**Table 1:** Demographics and baseline characteristics

Baseline characteristics (at the time of the switch)	
No. of patients	44
No. of eyes	57
Male, n (%)	30 (68.2)
Age (years), mean (range)	64.4 (41–79)
Type 2 diabetes, n (%)	39 (88.6)
Right eyes, n (%)	28 (49.1)
Phakic, n (%)	45 (78.9)
Baseline BCVA (letters), median (IQR)	69 (62–89)
Baseline CMT (μm), median (IQR)	400 (358–451)
No. of ranibiumab injections, mean (range)	12 (3- 21)
Duration of ranibizumab treatment (months), mean (range)	17.9 (2.7–42.3)
Interval between last ranibizumab and first aflibercept (days), mean (range)	62.9 (28–119)
No. of eyes with other treatments before first ranibizumab, n (%)	
Focal/grid laser	28 (49.1)
Panretinal photocoagulation	17(29.8)
Triamcinolone	5(8.8)
No. of eyes with other treatments during treatment with ranibizumab, n (%)	
Focal/grid laser	6(10.5)
Panretinal photocoagulation	9(15.8)
Vitrectomy	1(1.7)

### Data capture

The mean follow-up period after treatment switch was 36.7 (34.7–38.43) months. The aim was to capture data every 3 months (including 3 and 6 months before treatment switch). Due to the retrospective nature of the study, actual follow-up dates did not always coincide with the desired follow-up date. The median (IQR) difference between the two dates was 0.0 days (−21–20). For simplicity the follow-up period is reported in trimesters.

### Initial response to ranibizumab

Median duration of ranibizumab treatment was 17.9 (range 2.7–42.3) months, during which the eyes received a mean number of 12 injections (range 3–21). The median (IQR) CMT improved from 461 (417–523) μm before treatment with ranibizumab, to 402 (345–476) μm at 6 months and to 374 (328–431) μm at 3 months prior to switch. The median (IQR) BCVA improved from 66 (58–74) letters before treatment with ranibizumab, to 70 (62–77) letters at 6 months and to 74 (64–77) letters at 3 months prior to switch. However, at the date when the switch to aflibercept was deemed necessary, the CMT had increased to 400 (358–451) μm and the vision had decreased to 69 (62–74) letters.

### Visual outcomes

**Fig. 1:**
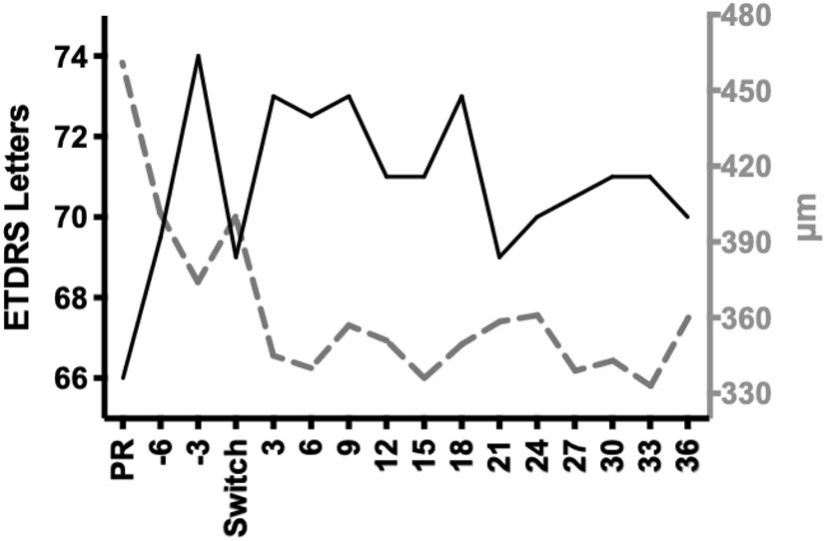
Median best corrected visual acuity in letters (solid line) and median central macular thickness in μm (dashed line) before the initiation of treatment with ranibizumab (PR), 6 and 3 months before the switch to aflibercept, at the day of the decision to switch and every 3 months after the switch up to 36 months follow-up

Following switch to aflibercept, median (IQR) BCVA improved to 73 (64–77) letters at 3 months (*p* = 0.0006), to 73 (61–78) letters at 6 months (p = 0.0042), to 73 (65–77) at 9 months (*p* = 0.0006), and to 73 (63–75) letter at 18 months (p = 0.0444). A statistically significant difference was not noted for the following trimesters up to 36 months, compared to the date of the switch (Wilcoxon matched-pairs signed rank test, n_3m_ = 57, n_6m_ = 56, n_9m_ = 55, n_18m_ = 57) (Table 2 and Fig. 1). At 36 months following switch, 12 eyes had gained more than 10 letters of vision, 5 eyes had gained 5–9 letters, 25 remained stable (gained or lost less than 5 letters), 7 eyes lost 5–9 letters and 8 eyes lost more than 10 letters (Additional file 1: Table S1).

**Table 2:** Median BCVA at each visit

BCVA								
Time point	Pre ranibizumab	6 m pre switch	3 m pre switch	Baseline (switch)	3 m post switch	6 m post switch	9 m post switch	12 m post switch
Median (IQR) BCVA in letters	66 (58–74)	70 (62–77)	74 (64–77)	69 (62–74)	73 (64–77)	73 (61–78)	73 (65–77)	71 (59–77)
n	54	54	54	57	57	56	55	57
Statistical significance	ns	ns	**		***	**	***	ns
p value	0.3614	0.3314	0.0051		0.0006	0.0042	0.0006	0.066

BCVA								
Time point	15 m post switch	18 m post switch	21 m post switch	24 m post switch	27 m post switch	30 m post switch	33 m post switch	36 m post switch
Median (IQR) BCVA in letters	71 (61–75)	73 (62–75)	69 (61–75)	70 (60–74)	71 (59–75)	71 (65–75)	71 (65- 78)	70 (64–77)
n	56	57	57	57	54	57	53	57
Statistical significance	ns	*	ns	ns	ns	ns	ns	ns
p value	0.0972	0.0444	0.4118	0.5681	0.8975	0.1288	0.143	0.3168

Best corrected visual acuity (BCVA) before the initiation of treatment with ranibizumab, 6 and 3 months before the switch to aflibercept, at the day of the decision to switch and every 3 months after the switch up to 36 months follow-up. BCVA was significantly improved compared to baseline 3 months pre switch, 3, 6, 9 and 18 months post switch (Wilcoxon matched-pairs signed rank test)

### Anatomical outcomes

Switch of treatment from ranibizumab to aflibercept led to a statistically significant reduction in CMT at all trimesters of follow-up except at month 24. More specifically, median (IQR) CMT improved from 400 (358–451) μm at the date of the switch to 345 (315–365) μm at 3 months (p < 0.0001), to 340 (314–391) μm at 6 months (p < 0.0001), to 351 (314–397) μm at 12 months (p = 0.0011), and to 360 (310–411) μm at 36 months (p = 0.0107), (Wilcoxon matched-pairs signed rank test, n_3m_ = 55, n_6m_ = 55, n_12m_ = 51, n_36m_ = 57) (Table 3 and Fig. 1). At 36 months, 30 eyes had more than 50 μm reduction in CMT, 17 eyes remained stable (less than 50 μm reduction or gain) and 10 eyes had more than 50 μm increase in CMT since the switch (Additional file 2: Table S2).

**Table 3:** Median CMT at each visit

CMT								
Time point	Pre ranibizumab	6 m pre switch	3 m pre switch	Baseline (switch)	3 m post switch	6 m post switch	9 m post switch	12 m post switch
Median (IQR) CMT in μm	461 (417–523)	402 (345–476)	374 (328–431)	400 (358–451)	345 (315–365)	340 (314–391)	357 (321–395)	351 (314–397)
n	57	54	52	57	55	55	49	51
Statistical significance	****	ns	*		****	****	***	**
p value	< 0.0001	0.9404	0.0439		< 0.0001	< 0.0001	0.0004	0.0011

CMT								
Time point	15 m post switch	18 m post switch	21 m post switch	24 m post switch	27 m post switch	30 m post switch	33 m post switch	36 m post switch
Median (IQR) CMT in μm	336 (308- 378)	350 (320- 416)	359 (323- 385)	361 (307- 432)	339 (318- 387)	343 (302- 397)	333 (307- 395)	360 (310- 411)
n	52	52	48	41	37	50	40	57
Statistical significance	****	***	**	ns	***	****	***	*
p value	< 0.0001	0.0004	0.0039	0.21	0.0003	< 0.0001	0.0003	0.0107

Central macular thickness (CMT) before the initiation of treatment with ranibizumab, 6 and 3 months before the switch to aflibercept, at the day of the decision to switch and every 3 months after the switch up to 36 months follow-up. CMT was significantly decreased compared to baseline 3 months pre switch and every trimester post switch except in month 24. (Wilcoxon matched-pairs signed rank test)

### Number of injections

The mean number of ranibizumab injections was 12 (range 3–21) over a mean period of 17.9 (range 12.4–42.3) months. The mean number of aflibercept injections during the 36 months of follow-up was 14 (range 3- 22) over a mean period of 36.7 (range 34.7–38.4) months (Table 4).The mean number of injections per month was significantly reduced from 0.76 (range 0.37–1.4) with ranibizumab, to 0.38 (range 0.082–0.57) injections with aflibercept (*p* < 0.0001, paired t-test).

**Table 4:** Number of injections

Number of injections								
Time point	6 m pre switch	3 m pre switch	Baseline (switch)	3 m post switch	6 m post switch	9 m post switch	12 m post switch	15 m post switch
Mean number of injections (min- max)	8.6 (0–18)	11 (2–20)	12 (3–21)	2.2 (1–3)	3.6 (1–6)	4.8 (2–7)	5.9 (3–8)	7.3 (3–11)
n	57	57	57	57	57	57	57	57

Time point	18 m post switch	21 m post switch	24 m post switch	27 m post switch	30 m post switch	33 m post switch	36 m post switch
Mean number of injections (min- max)	8.4 (3–13)	9.3 (3–14)	10 (3–15)	11 (3–16)	12 (3–18)	13 (3–20)	14 (3–22)
n	57	57	57	57	57	57	57

Mean (range) number of ranibizumab injections 6 and 3 months before the switch to aflibercept, at the day of the decision to switch and mean (range) number of aflibercept injections every 3 months after the switch up to 36 months follow-up

### Further treatment and adverse events

During treatment with ranibizumab, 6 eyes received focal/grid laser, 9 eyes PRP, and 1 eye underwent combined PPV with internal limiting membrane (ILM) peel and cataract surgery. During treatment with aflibercept, 1 eye received focal/grid laser, 5 eyes PRP, 7 eyes had cataract surgery and 1 had posterior capsulotomy.

One eye was treated for endophthalmitis with PPV and intravitreal antibiotics. The same eye subsequently underwent PPV, PRP endolaser, epiretinal membrane (ERM) peel and cataract surgery. Two eyes underwent combined PPV with ILM and ERM peel, and cataract surgery for ERM.

One eye had tractional retinal detachment treated with PPV, ILM and ERM peel, proliferative vitreoretinopathy peel, and cataract surgery. Three eyes developed vitreous haemorrhage, two of which were treated with PPV. There was no difference noted in outcome in eyes who received previous laser (grid/pan retinal photocoagulation/vitrectomy/steroid) before initiation of anti-VEGF therapy or top up during anti-VEGF therapy.

No systemic adverse events were recorded during the study period.

## Discussion

DMO is the leading cause of vision loss in diabetic patients. Previously, laser was considered the gold standard treatment of these patients based on the landmark Early Treatment Diabetic Retinopathy Group and on the Diabetic Retinopathy Study trials [17, 18].The advent of anti-VEGF treatment has led to a major shift in both the management and prognosis of DMO.

Although anti-VEGF injections have been shown to improve both anatomical and functional outcomes in patients with DMO, a proportion of patients demonstrate a sub-optimal response [4–7]. Options faced by clinicians in such circumstances include intravitreal corticosteroids or trialling a different anti-VEGF agent. With a comparatively lower side-effect profile, particularly in a younger population, trialling an alternative anti-VEGF agent is often considered preferable to corticosteroids.

In our cohort the median BCVA improved at 3, 6, 9 and 18 months following switch and 42 eyes (73.7%) gained vision or remained stable at 36 months. Furthermore, median CMT was noted to be significantly reduced in all trimesters of follow-up with the exception of the 24th month with only 10 eyes (17.5%) having increase of more than 50 μm at the end of follow-up.

Our data suggests sustained improvement in both CMT and BCVA at 36 months following treatment switch from ranibizumab to aflibercept. These results are consistent with several previous studies with a shorter duration of follow-up [8–11, 16, 19–21]. Other studies have demonstrated anatomical improvement following treatment switch, yet no associated improvement in vision [12–15].

Data from Protocol T suggests a greater benefit of aflibercept when compared with ranibizumab or bevacizumab for eyes treated for DMO with a lower baseline BCVA [4]. Furthermore, less patients treated with aflibercept required additional laser photocoagulation [4]. Secondary analysis of the results showed that in year 1 and 2, eyes with pre-proliferative retinopathy receiving anti-VEGF for DMO may experience improvement in retinopathy severity and all 3 agents were associated with low rates of DR progression. In a small subgroup analysis of patients receiving anti-VEGF injections for DMO, those receiving aflibercept demonstrated lower rates of DR regression compared with ranibizumab and bevacizumab in eyes with proliferative diabetic retinopathy at baseline [6, 7].

A potential benefit of aflibercept compared with ranibizumab in the treatment of DMO may relate to the differing structural, pharmacokinetic and pharmacodynamic properties. Aflibercept has a markedly higher affinity for VEGF-A than bevacizumab or ranibizumab and additionally binds to VEGF-B and placental growth factor [22]. The latter is a cytokine that can stimulate angiogenesis and plays a crucial role in the activation and maintenance of the inflammatory switch associated with neo-angiogenesis. Placental growth factor has been implicated in the pathogenesis of diabetic retinopathy and DMO [23].

Another potential mechanism for a greater improvement with aflibercept might be related to tachyphylaxis or diminished therapeutic response over time after repetitive injections of ranibizumab [24].

Our study lacks a control group of patients for comparison and it therefore remains possible that continued treatment with ranibizumab, regardless of initial response, may have led to improved CMT and BCVA over time. In the RIDE and RISE studies, 9–10% of eyes treated with ranibizumab demonstrated a delayed response, with visual acuity gains and diabetic retinopathy improvement similar to the eyes that had immediate anatomic response [25, 26]. Furthermore, recent data from Protocol T suggests that some patients with suboptimal anti-VEGF response at 12 weeks experienced improved BCVA at 2 years without switching anti-VEGF agents, supporting the possibility of a delayed response in some patients [27].

Thus, controversy remains around the optimal timing of switching anti-VEGF agents. Some advocate early switching as prolonged DMO can lead to permanent structural damage to the neural retina, hindering visual gain, as seen in the delayed anti-VEGF treatment arms of the RISE/RIDE and VISTA/VIVID trials [28, 29]. In contrast, others advocate delaying the decision to switch anti-VEGF agents due to the possibility of late response [30].

Our study is limited by its retrospective design. During the represented time period, there was no protocol regarding which eyes should undergo treatment switch and when this should occur in our department. As such, the number of previous ranibizumab injections prior to treatment switch ranged from 3 to 21. Furthermore, as previously mentioned, we lack a control group for a true comparison of outcomes between those switching to aflibercept, and those remaining on ranibizumab treatment.

Despite the intrinsic limitations of this study, we provide real world data with the longest follow-up period to date suggesting a sustained anatomical and functional benefit of switching from ranibizumab to aflibercept in the treatment of refractory DMO. Further studies with larger numbers of patients are required in order to identify the subset of patients that may benefit from treatment switch, as well as the ideal time of switching treatment.

## Supplementary Information

**Additional file 1: Table S1.** Number of eyes per visual gain or loss category. Number of eyes gaining more than 10 letters, gaining 5–9 letters, remaining stable (gaining or losing less than 5 letters), losing 5–9 letters and losing more than 10 letters every trimester after the switch, up to 36 months follow-up.

**Additional file 2: Table S2. **Number of eyes per CMT increase or reduction category. Number of eyes with more than 50 μm reduction in CMT, remaining stable (increase or decrease less than 50 μm), more than 50 μm increase in CMT every trimester after the switch, up to 36 months follow-up.

## Data Availability

Anonymized data are available from the corresponding author on reasonable request.

## Abbreviations

DMO: Diabetic macular oedema
NICE: National Institute for Clinical Excellence
CRT: Central retinal thickness
BCVA: Best corrected visual acuity
PPV: Pars plana vitrectomy
PRP: Panretinal photocoagulation
ILM: Internal limiting membrane
ERM: Epiretinal membrane
