# Supplementary material for: Functional and structural characteristics in patients with diabetic macular oedema after switching from ranibizumab to aflibercept treatment. Three year results in real world settings
Source: Int J Retina Vitreous. 2022 Apr 1;8:23. doi: 10.1186/s40942-022-00373-5 (PMC8973508; doi:10.1186/s40942-022-00373-5)
Supplement: Supplementary file 1 — Additional file 1: Table S1. Number of eyes per visual gain or loss category. Number of eyes gaining more than 10 letters, gaining 5–9 letters, remaining stable (gaining or losing less than 5 letters), losing 5–9 letters and losing more than 10 letters every trimester after the switch, up to 36 months follow-up. [file 40942_2022_373_MOESM1_ESM.docx]

**Table S1. Number of eyes per visual gain or loss category**

Number of eyes gaining more than 10 letters, gaining 5-9 letters, remaining stable (gaining or losing less than 5 letters), losing 5-9 letters and losing more than 10 letters every trimester after the switch, up to 36 months follow-up.

| **Letters** | **3 m post switch** | **6 m post switch** | **9 m post switch** | **12 m post switch** | **15 m post switch** | **18 m post switch** | **21 m post switch** | **24 m post switch** | **27 m post switch** | **30 m post switch** | **33 m post switch** | **36 m post switch** |
| --- | --- | --- | --- | --- | --- | --- | --- | --- | --- | --- | --- | --- |
| >10 gained | 8 | 7 | 9 | 8 | 10 | 9 | 9 | 10 | 10 | 12 | 14 | 12 |
| 5 to 9 | 11 | 10 | 15 | 12 | 10 | 13 | 9 | 13 | 7 | 5 | 5 | 5 |
| <5 lost or gained | 31 | 32 | 24 | 25 | 24 | 24 | 27 | 18 | 25 | 29 | 21 | 25 |
| 5 to 9 lost | 7 | 4 | 5 | 7 | 7 | 6 | 4 | 6 | 4 | 5 | 10 | 7 |
| more than 10 loss | 0 | 3 | 2 | 5 | 5 | 5 | 8 | 10 | 8 | 6 | 3 | 8 |
| Total number | 57 | 56 | 55 | 57 | 56 | 57 | 57 | 57 | 54 | 57 | 53 | 57 |
