# Supplementary material for: Functional and structural characteristics in patients with diabetic macular oedema after switching from ranibizumab to aflibercept treatment. Three year results in real world settings
Source: Int J Retina Vitreous. 2022 Apr 1;8:23. doi: 10.1186/s40942-022-00373-5 (PMC8973508; doi:10.1186/s40942-022-00373-5)
Supplement: Supplementary file 2 — Additional file 2: Table S2. Number of eyes per CMT increase or reduction category. Number of eyes with more than 50 μm reduction in CMT, remaining stable (increase or decrease less than 50 μm), more than 50 μm increase in CMT every trimester after the switch, up to 36 months follow-up. [file 40942_2022_373_MOESM2_ESM.docx]

**Table S2. Number of eyes per CMT increase or reduction category**

Number of eyes with more than 50μm reduction in CMT, remaining stable (increase or decrease less than 50μm), more than 50μm increase in CMT every trimester after the switch, up to 36 months follow-up.

| **CMT** | **3 m post switch** | **6 m post switch** | **9 m post switch** | **12 m post switch** | **15 m post switch** | **18 m post switch** | **21 m post switch** | **24 m post switch** | **27 m post switch** | **30 m post switch** | **33 m post switch** | **36 m post switch** |
| --- | --- | --- | --- | --- | --- | --- | --- | --- | --- | --- | --- | --- |
| >50μm reduction | 27 | 26 | 22 | 21 | 26 | 24 | 21 | 15 | 18 | 23 | 20 | 30 |
| <50 reduction or increase | 26 | 24 | 22 | 23 | 21 | 23 | 18 | 16 | 16 | 22 | 16 | 17 |
| >50μm increase | 2 | 5 | 5 | 7 | 5 | 5 | 9 | 10 | 3 | 5 | 4 | 10 |
| Total number | 55 | 55 | 49 | 51 | 52 | 52 | 48 | 41 | 37 | 50 | 40 | 57 |
